# Letter to the editor regarding “vitamin D supplementation decrease asthma exacerbations in children: a systematic review and meta-analysis of randomized controlled trials”

**DOI:** 10.1080/07853890.2025.2552927

**Published:** 2025-08-31

**Authors:** Usama Chaudhary, Zain Ul Abideen, Syeda Sunaina Hassan Gardezi

**Affiliations:** Nishtar Medical University, Multan, Pakistan

**Keywords:** Vitamin D, Asthma, Cancer, Vitamin D supplementation

Dear Editor,

Vitamin D is a broad term for two biologically active compounds: vitamin D_2_ (ergocalciferol), which is produced when ultraviolet B acts on ergosterol, and vitamin D_3_ (cholecalciferol), which is synthesized when 7-dehydrocholesterol present in human and animal skin is exposed to UVB. It plays an essential role in maintaining healthy bones, and its long-term deficiency can cause rickets in children and osteomalacia in adults [1].

Evidence suggests that vitamin D influences both innate and adaptive immune responses, which seem especially relevant to allergic and chronic respiratory diseases. Vitamin D deficiency has been linked to an increased risk of developing chronic and progressive lung diseases. Supplementation with vitamin D has shown benefits in reducing these risks and improving respiratory health. “*Vitamin D supplementation decreases asthma exacerbations in children: a systematic review and meta-analysis of randomized controlled trials*’’ *by Fedora* et al. concluded that vitamin D supplementation can reduce the occurrence of asthma exacerbations in children and improve lung function, i.e., forced expiratory volume in 1 s (FEV_1_), by diminishing Th2 and Th17 responses, intensifying IL-10 production, and hampering airway smooth muscle hypertrophy and collagen deposition [2]. However, these findings are inconsistent, as a recently published article by Li et al. titled “*The effects of prenatal vitamin D supplementation on respiratory and allergy-related outcomes in children: A systematic review and meta-analysis of randomized controlled trials*” [3] highlighted that vitamin D supplementation during pregnancy may not significantly lower the risk of respiratory infections and allergy-related outcomes in children. Therefore, high-quality large-scale randomized controlled trials are required to better understand the benefits and safety of vitamin D supplementation in adults and children with asthma and vitamin D deficiency.

Moreover, vitamin D supplementation has recently been explored as a potential strategy for cancer prevention. Preclinical and observational data suggest that low levels of 25-hydroxyvitamin D [25(OH)D] may be linked to a higher risk of developing various site-specific cancers, including those of the colon, breast, bladder, lung, pancreas, prostate, and certain childhood cancers. However, vitamin D supplementation is found to reduce mortality in patients with lung cancer only [4]. Endometrial adenocarcinoma is the most common invasive tumor of the female reproductive system, primarily affecting postmenopausal women. Studies have shown that low doses (10^−9^–10^−11^ M) of vitamin D can inhibit the multiplication and relocation of endometrial adenocarcinoma (HEC1A) cells. However, at higher concentrations (10^−6^–10^−8^ M), vitamin D may have the opposite effect, promoting cell growth and partially enhancing migration [5]. Vitamin D treatment can induce cell cycle arrest in endometrial cancer cells by suppressing some regulators of cell cycle progression, like cyclin D1 and cyclin D3, and enhancing the expression of p27, a well-known cell cycle inhibitor, with an additive effect when used in combination with progesterone. Considering these findings, further research is necessary to fully understand vitamin D’s role in cancer and establish clear clinical guidelines. In addition, vitamin D’s role in asthma and other respiratory diseases should be fully known, and further clinical research is required in this regard.

## Data Availability

Data sharing does not apply to this article as no data was created or analyzed in this study.
